# Transcriptional regulation of flavonol biosynthesis in plants

**DOI:** 10.1093/hr/uhae043

**Published:** 2024-02-15

**Authors:** Yunlin Cao, Yuyang Mei, Ruining Zhang, Zelong Zhong, Xiaochun Yang, Changjie Xu, Kunsong Chen, Xian Li

**Affiliations:** Zhejiang Provincial Key Laboratory of Horticultural Crop Quality Manipulation, Zhejiang University, Hangzhou, 310058, China; Center for Drug Safety Evaluation and Research, College of Pharmaceutical Sciences, Zhejiang University, Hangzhou, 310058, China; Shandong (Linyi) Institute of Modern Agriculture, Zhejiang University, Linyi, 276000, China; Zhejiang Provincial Key Laboratory of Horticultural Crop Quality Manipulation, Zhejiang University, Hangzhou, 310058, China; Zhejiang Provincial Key Laboratory of Horticultural Crop Quality Manipulation, Zhejiang University, Hangzhou, 310058, China; College of Food Science and Nutritional Engineering, China Agricultural University, Beijing 100083, China; Center for Drug Safety Evaluation and Research, College of Pharmaceutical Sciences, Zhejiang University, Hangzhou, 310058, China; Shandong (Linyi) Institute of Modern Agriculture, Zhejiang University, Linyi, 276000, China; Zhejiang Provincial Key Laboratory of Horticultural Crop Quality Manipulation, Zhejiang University, Hangzhou, 310058, China; Zhejiang Provincial Key Laboratory of Horticultural Crop Quality Manipulation, Zhejiang University, Hangzhou, 310058, China; Zhejiang Provincial Key Laboratory of Horticultural Crop Quality Manipulation, Zhejiang University, Hangzhou, 310058, China; Shandong (Linyi) Institute of Modern Agriculture, Zhejiang University, Linyi, 276000, China

## Abstract

Flavonols are a class of flavonoids that play a crucial role in regulating plant growth and promoting stress resistance. They are also important dietary components in horticultural crops due to their benefits for human health. In past decades, research on the transcriptional regulation of flavonol biosynthesis in plants has increased rapidly. This review summarizes recent progress in flavonol-specific transcriptional regulation in plants, encompassing characterization of different categories of transcription factors (TFs) and microRNAs as well as elucidation of different transcriptional mechanisms, including direct and cascade transcriptional regulation. Direct transcriptional regulation involves TFs, such as MYB, AP2/ERF, and WRKY, which can directly target the key *flavonol synthase* gene or other early genes in flavonoid biosynthesis. In addition, different regulation modules in cascade transcriptional regulation involve microRNAs targeting TFs, regulation between activators, interaction between activators and repressors, and degradation of activators or repressors induced by UV-B light or plant hormones. Such sophisticated regulation of the flavonol biosynthetic pathway in response to UV-B radiation or hormones may allow plants to fine-tune flavonol homeostasis, thereby balancing plant growth and stress responses in a timely manner. Based on orchestrated regulation, molecular design strategies will be applied to breed horticultural crops with excellent health-promoting effects and high resistance.

## Introduction

**Figure 1:**
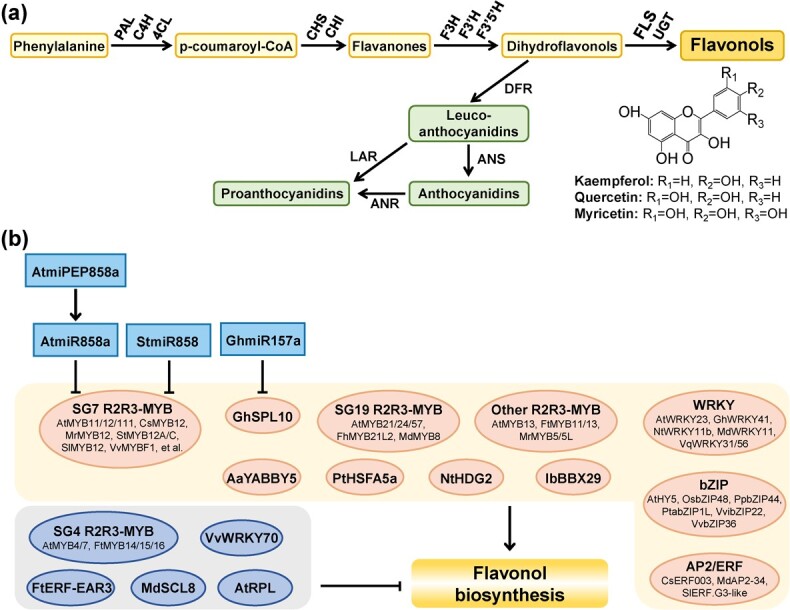
The phenylpropanoid pathway and direct transcriptional and post-transcriptional regulation of flavonol biosynthesis in plants. **a** The phenylpropanoid pathway leading to the biosynthesis of flavonols and other flavonoids. PAL, phenylalanine ammonia lyase; C4H, cinnamate 4-hydroxylase; 4CL, 4-coumaroyl CoA ligase; CHS, chalcone synthase; CHI, chalcone isomerase; F3H, flavanone 3-hydroxylase; F3′H, flavonoid 3′-hydroxylase; F3′5′H, flavonoid 3′5′-hydroxylase; FLS, flavonol synthase; UGT, uridine diphosphate-glycosyltransferases; DFR, dihydroflavonol 4-reductase; ANS, anthocyanidin synthase; LAR, leucoanthocyanidin reductase; ANR, anthocyanidin reductase. **b** Direct transcriptional and post-transcriptional regulation of flavonol biosynthesis in plants. Light yellow and light gray backgrounds indicate activators and repressors, respectively. Light sky-blue rectangles represent microRNAs; light orange and blue-gray ovals represent activators and repressors, respectively.

Flavonols belong to one class of flavonoids with the C_6_-C_3_-C_6_ basic structure and are characterized by the carbon–carbon bond (C2 and C3 positions), a hydroxyl group (C3 position), and a carbon group (C4 position) in the heterocyclic C-ring (Fig. 1a). Due to the varying substitutions of hydroxylation and methoxylation on the A and B rings, flavonol aglycones can be classified into more than 10 different types [1]. Among them, kaempferol, quercetin, and myricetin are the most prevalent flavonol aglycones in the plant kingdom [1, 2] (Fig. 1a). Flavonols are commonly found in glycosylated forms and are ubiquitously distributed in various plant tissues [1, 2]. They play a vital role in plant development and stress resistance, including regulating auxin transport, affecting root and pollen development, influencing pollinator preference and reproductive isolation, UV-B protection, and enhancing disease resistance [3–8].

Moreover, numerous studies have reported that flavonols have strong antioxidant activity and play important roles in preventing chronic non-communicable diseases such as cancers, cardiovascular diseases, and diabetes in humans [9–14]. In several representative staple crops, such as potato (*Solanum tuberosum*), barley (*Hordeum vulgare*), corn (*Zea mays*), and wheat (*Triticum aestivum*), the flavonol content of their edible part is <2 mg/100 g fresh or dry weight [15–18] (Table 1). The medicinal value of medicinal plants, including *Ginkgo biloba* and Tartary buckwheat (*Fagopyrum tataricum*), is considered to be closely related to their high contents of flavonols [19–21]. In addition, the flavonol content of horticultural plants, such as tea (*Camellia sinensis*), onion (*Allium cepa*), asparagus (*Asparagus officinalis*), Chinese cabbage (*Brassica rapa* var. *chinensis*), berries, apple (*Malus × domestica*), tomato (*Solanum lycopersicum*), broccoli (*Brassica oleracea* var. *botrytis*), peppers (*Capsicum annuum*), citrus (*Citrus sinensis*), kiwifruit (*Actinidia chinensis*), mango (*Mangifera indica*), and grape (*Vitis vinifera*), ranged from 2 to 2727 mg/100 g fresh weight and was associated with their health-promoting effects [22–31] (Table 1). Therefore, horticultural crops and medicinal and edible plants may be the main contributors to people’s daily dietary intakes of flavonols.

**Table 1:** Flavonol content in the edible portion of staple crops, medicinal plants, and horticultural plants.

**Species**	**Content (mg/100 g FW)**	**Reference**	**Species**	**Content (mg/100 g FW)**	**Reference**
Tea	2727	22	Chinese bayberry	3–66	26
*Ginkgo biloba*	176	19, 20	Apple, peel	22–35	29
Tartary Buckwheat	1–175^*^	21	Cranberry	16–26	27
Onion	49–98	23	Crowberry	10	27
Asparagus	24–86	24	Cherry	10	28
Chinese cabbage	49	25	Chokeberry	9	27
Mugwort	43	25	Papaya	7	28
Parsley	27	30	Citrus	6	30
Tomato, peel	18	31	Blueberry	4–5	27
Broccoli	18	25	Kiwifruit	3	30
Pepper	12	25	Strawberry	2	30
Lettuce	8	25	Mango	2	28
Cauliflower	5	25	Grape	2	30
Snap bean	4	30	Potato, tuber	2*	15
Broccoli	3	30	Barley, flour	2*	16
Carrot	3	25	Corn	<1	17
*Salvia officinalis*	n.d.	19	Wheat	n.d.	18

Flavonols in plants are usually present in glycosylated forms. Therefore, the flavonol content was defined as the total content of flavonol glycosides. *Dry weight basis; FW, fresh weight; n.d., not detected.

Until now, more than 26 000 articles can be retrieved from the PubMed database (https://pubmed.ncbi.nlm.nih.gov/) by using the keyword ‘quercetin’, one class of flavonol aglycone. In the past few decades, significant progress has been made in understanding the transcription factors (TFs) and microRNAs (miRNAs) that regulate flavonol biosynthesis in plants. However, to date there has been no systematic review of the transcriptional regulation of flavonol biosynthesis in plants.

This review focuses on recent advances in knowledge and understanding of the transcriptional regulation of flavonol biosynthesis in plants. Progress in the characterization of TFs and miRNAs regulating flavonol biosynthesis will be summarized, with particular emphasis on direct transcriptional regulation and cascade transcriptional regulation of flavonol biosynthesis. Direct transcriptional regulation involves TFs, such as MYB, AP2/ERF, and WRKY, which directly target the key *FLS* gene and other early genes in the flavonoid biosynthesis pathway. In addition, different regulation modules in cascade transcriptional regulation will be summarized and discussed; these consist of miRNAs targeting TFs, regulation between activators, interaction between activators and repressors, and degradation of activators or repressors induced by environmental signals such as UV-B or plant hormones. This review may provide valuable insights for the production of horticultural crops with a high content of flavonols that are beneficial for human health.

## Flavonol biosynthesis pathway

The flavonol biosynthesis pathway is clear now in plants [32] (Fig. 1a). Phenylalanine is catalyzed to form *p*-coumaroyl-CoA under the action of phenylalanine ammonia lyase (PAL), cinnamate 4-hydroxylase (C4H), and 4-coumaroyl CoA ligase (4CL), which make up the general phenylpropanoid pathway. Chalcone synthase (CHS) is the first enzyme of flavonoid biosynthesis and acts on *p*-coumaroyl-CoA as a substrate to synthesize chalcone, which is then acted on by chalcone isomerase (CHI) to yield naringenin. Naringenin is further hydroxylated by flavanone 3-hydroxylase (F3H), flavonoid 3′-hydroxylase (F3′H), or flavonoid 3′5′-hydroxylase (F3′5′H) to produce dihydroflavonols with different hydroxylation patterns [33]. The enzymes, including CHS, CHI, F3H, F3′H, and F3′5′H, comprise the early steps of the flavonoid biosynthetic pathway. Furthermore, dihydroflavonols can be oxidized by flavonol synthase (FLS) to form flavonol aglycones, such as kaempferol, quercetin, or myricetin [33]. Flavonol aglycones finally undergo modifications by uridine diphosphate-glycosyltransferases (UGTs), *O*-methyltransferases (OMTs), and acyltransferases (ATs), leading to the formation of various and stable flavonol derivatives [34–36]. In addition, dihydroflavonols also can be acted on by dihydroflavonol 4-reductase (DFR) to produce leucoanthocyanidins and to direct flavonoid metabolism to the synthesis of anthocyanins and proanthocyanidins. The competition between FLS and DFR regulates metabolic flux to different branches of the flavonoid biosynthetic pathway [37].

**Table 2:** Different types of transcription factors or regulation modules involved in regulating flavonol biosynthesis in plants.

**Transcription factor**	**Species**	**Representative genes**	**Function**	**References**
SG7 R2R3-MYB	*Arabidopsis thaliana*, *Camellia sinensis*, *Cicer arietinum*, *Citrus sinensis*, *Cucumis sativus*, *Cynara cardunculus*, *Epimedium sagittatum*, *Erigeron breviscapus*, *Freesia hybrida*, *Fagopyrum tataricum*, *Gentiana triflora*, *Gerbera hybrida*, *Malus* (crab apple), *Malus × domestica*, *Medicago truncatula*, *Morella rubra*, *Muscari aucheri*, *Nicotiana tabacum*, *Paeonia qiui*, *Paeonia suffruticosa*, *Petunia axillaris*, *Prunus persica*, *Pyrus bretschneideri*, *Pyrus pyrifolia*, *Solanum lycopersicum*, *Solanum tuberosum*, *Vitis vinifera*	*AtMYB11/12/111*, *CsMYB12*, *CaMYB39*, *CsMYBF1*, *CsMYB60*, *CcMYB12*, *EsMYBF1*, *EbMYBP1*, *FhMYBF1/2/3/4*, *FtMYB6*, *GtMYBP3/4*, *GhMYB1a*, *McMYB4*, *MdMYB22*, *MtMYB134*, *MrMYB12*, *MaMYBF*, *NtMYB12/184*, *PqMYBF1*, *PsMYB111*, *PaMYB-FL*, *PpMYB15*, *PpMYBF1*, *PbMYB12b*, *PpMYB17*, *SlMYB12*, *StMYB12A/C*, *VvMYBF1*	Activator	8, 15, 37, 42, 43, 44, 45, 46, 47, 48, 49, 50, 51, 52, 53, 54, 55, 56, 57, 58, 59, 60, 61, 62, 63, 64, 65, 66, 67, 68, 69, 70
SG19 R2R3-MYB	*Arabidopsis thaliana*, *Freesia hybrida*, *Malus* (crab apple)	*AtMYB21/24/57*, *FhMYB21L2*, *MdMYB8*	Activator	46, 71, 72
SG4 R2R3-MYB	*Arabidopsis thaliana*, *Fagopyrum tataricum*	*AtMYB4/7*, *FtMYB14/15/16*	Repressor	73, 74, 75
Other R2R3-MYB	*Arabidopsis thaliana*, *Fagopyrum tataricum*, *Morella rubra*	*AtMYB13*, *FtMYB11/13*, *MrMYB5*/*5L*	Activator	7, 47, 74, 76
WRKY	*Arabidopsis thaliana*, *Gossypium hirsutum*, *Malus domestica*, *Nicotiana tabacum*, *Vitis quinquangularis*, *Vitis vinifera*	*AtWRKY23*, *GhWRKY41*, *NtWRKY11b*, *MdWRKY11*, *VqWRKY31/56*, *VvWRKY70*	Activator or repressor	5, 77, 78, 79, 80, 81, 82
bZIP	*Arabidopsis thaliana*, *Oryza sativa*, *Pyrus pyrifolia*, *Populus tremula × P. alba*, *Vitis vinifera*	*AtHY5*, *OsbZIP48*, *PpbZIP44*, *PtabZIP1L*, *VvibZP22*, *VvbZIP36*	Activator	83, 84, 85, 86, 87, 88
AP2/ERF	*Citrus sinensis*, *Fagopyrum tataricum*, *Malus domestica*, *Solanum lycopersicum*	*CsERF003*, *FtERF-EAR3*, *MdAP2-34*, *SlERF.G3-like*	Activator or repressor	89, 90, 91, 92
GRAS	*Malus domestica*	*MdSCL8*	Repressor	93
YABBY	*Artemisia annua*	*AaYABBY5*	Activator	94
HSF	*Populus tomentosa*	*PtHSFA5a*	Activator	95
BLH	*Arabidopsis thaliana*	*AtRPL*	Repressor	96
HD-ZIP	*Nicotiana tabacum*	*NtHDG2*	Activator	97
BBX	*Ipomoea batatas*	*IbBBX29*	Activator	98

## Direct transcriptional regulation of flavonol biosynthesis

### R2R3-MYB transcription factors

The MYB (V-myb avian myeloblastosis viral oncogene homolog) family is widely present in all eukaryotes and represents one of the largest TF families in plants. The N-terminus of MYB family proteins contains a highly conserved DNA-binding domain which typically consists of one to four imperfect repeats [38]. Each repeat of ~50–53 amino acids forms a helix–turn–helix structure, allowing them to bind to the major groove of the DNA double helix [39]. MYB proteins are classified into four types: MYB-related (R1/2-MYB, R3-MYB), R2R3-MYB, 3R-MYB (R1R2R3-MYB), and 4R-MYB [38, 40]. R2R3-MYB TFs are the largest class in plants and are composed of two DNA-binding repeats. Based on phylogenetic relationships and the presence of conserved motifs in the C-terminal region, R2R3-MYB proteins are classified into different subgroups, including subgroup 4 (SG4), SG7, SG19, etc. [38, 40, 41]. In past decades there has been an increasing number of studies focusing on R2R3-MYB TFs involved in regulating flavonol biosynthesis (Fig. 1b and Table 2).

*SG7 R2R3-MYBs*. The SG7 R2R3-MYBs are flavonol-specific regulators with the characteristic SG7 (GRTxRSxMK) motif and have been characterized in numerous plants. In *Arabidopsis thaliana*, AtMYB12, along with its homologs AtMYB11 and AtMYB111, belongs to SG7 of the R2R3-MYB family. These proteins controlled flavonol biosynthesis by independently activating expression of *AtCHS*, *AtCHI*, *AtF3H*, and *AtFLS1* [42, 43]. MdMYB22 from apple and CsMYB12 from tea are the homologs of AtMYB12 and have been shown to act as activators via binding to the *FLS* promoter and activating its transcription in *vivo*, as confirmed through yeast one-hybrid and luciferase assays [44, 45]. In *Freesia hybrida*, chromatin immunoprecipitation-quantitative polymerase chain reaction (ChIP–qPCR) and *β*-glucuronidase assays indicated that FhMYB1/2/3/4 bind to MYBCORE and AC-rich elements in promoters of *FhCHI2* and *FhFLS1* to activate their transcription [46]. Furthermore, MrMYB12 from Chinese bayberry (*Morella rubra*) was found to bind to the MYBCORE element in the *MrFLS2* promoter and activate its expression, as demonstrated by EMSA and luciferase assays [47]. These results demonstrate that SG7 R2R3-MYBs can directly target the key gene *FLS* and other early genes in the flavonoid biosynthetic pathway.

In addition, secondary metabolite profiling analyzed by LC–MS showed a selective reduction of glycosylated flavonol derivatives in single, double, or triple mutants of *Arabidopsis AtMYB11*, *AtMYB12*, and *AtMYB111* [43]. Meanwhile, the accumulation of other phenolic compounds in the mutant seedlings remained significantly unchanged [43]. In tomato, according to an LC–MS analysis, levels of 13 glycosylated flavonol derivatives, quercetin, naringenin, and naringenin chalcone were reduced in *pf* mutants with truncation of *SlMYB12* [48]. In *Petunia axillaris*, a *myb-fl* CRISPR mutant strongly reduced flavonol levels and expression of *FLS* and *HT1* (*F3′H*) [49]. In addition, overexpression of one SG7 *R2R3-MYB* gene, such as *AtMYB12* [37], *Gentiana triflora GtMYBP3/4* [50], *Epimedium sagittatum EsMYBF1* [51], peach (*Prunus persica*) *PpMYB15*/*PpMYBF1* [52], or *Chinese bayberry *MrMYB12** [53], resulted in flavonol accumulation in tobacco (*Nicotiana tabacum*) flowers. This was caused by upregulated expression of *NtFLS* and other early genes in the flavonoid biosynthesis pathway. Transgenic tobacco flowers changed from red to pale or pure white and showed a reduction in anthocyanin content, which was not consistent with the unaffected expression of *NtDFR* and other anthocyanin biosynthetic genes [37, 50, 52, 53]. Such apparent changes in flower color suggest that FLS may effectively compete with DFR to redirect the flux towards flavonol biosynthesis and away from anthocyanin biosynthesis. These findings indicate that SG7 R2R3-MYB TFs may be flavonol-specific activators in plants.

Subsequently, more SG7 R2R3-MYBs regulating flavonol biosynthesis were identified in other plant species, including citrus CsMYBF1 [54], chickpea (*Cicer arietinum*) CaMYB39 [8], cucumber (*Cucumis sativus*) CsMYB60 [55], *Cynara cardunculus* CcMYB12 [56], *Erigeron breviscapus* EbMYBP1 [57], Tartary buckwheat FtMYB6 [58], *Gerbera hybrida* GhMYB1a [59], *Malus* (crab apple) McMYB4 [60], *Medicago truncatula* MtMYB134 [61], *Muscari aucheri* MaMYBF [62], tobacco NtMYB12/184 [63–65], *Paeonia qiui* PqMYBF1 [66]*, Paeonia suffruticosa* PsMYB111 [67], *Pyrus bretschneideri* PbMYB12b [68], *Pyrus pyrifolia* PpMYB17 [69], potato StMYB12A/C [15], and grape VvMYBF1 [70].

High expression of SG7 *R2R3-MYB* genes may be one of reasons for high accumulation of flavonols in horticultural crops such as tea, apple, and Chinese bayberry (Table 1). In tea, high expression of *CsMYB12* in the first leaf or UV-B-irradiated leaf resulted in a high accumulation of quercetin glycosides and kaempferol glycosides [45]. In apple, expression of *MdMYB22* was positively correlated with the flavonol content in fruit of *F*_1_ hybrid populations of a cross between *Malus sieversii* f. *niedzwetzkyana* and *M. domestica* [44]. In Chinese bayberry, the transcript level of *MrMYB12* was induced by UV-B irradiation and was correlated with high accumulation of quercetin derivatives [47].

*SG19 R2R3-MYBs*. In addition to SG7 R2R3-MYBs, SG19 R2R3-MYB TFs were also found to be involved in the positive regulation of flavonol biosynthesis by activating transcription of the *FLS* gene. In *F. hybrida*, an SG19 R2R3-MYB protein, FhMYB21L2, could directly target and regulate one *FLS* member, *FhFLS2*, thus participating in flavonol accumulation in later developmental stages of the flower [46]. On the other hand, four SG7 R2R3-MYB members, namely FhMYBF1/2/3/4, were found to regulate expression of *FhFLS1* and flavonol biosynthesis in early developmental stages of the flower [46]. In *Arabidopsis*, SG19 R2R3-MYB members, including AtMYB21/24/57, have also been shown to be involved in regulating flavonol biosynthesis by controlling expression of *AtFLS1* [46, 71]. An SG19 R2R3-MYB TF, MdMYB8, was also identified in *Malus* crab apple and was responsible for quercetin 7-*O*-glucoside accumulation by regulating expression of *MdCHS* and *MdFLS* [72].

*Other R2R3-MYBs*. SG4 R2R3-MYB members contain an EAR motif (LNL[D/E]L) and have been identified as negative regulators of flavonols and other phenolic compounds. In *Arabidopsis*, the *atmyb4 atmyb7* mutant showed an increase in the accumulation of flavonols and anthocyanins, caused by induced expression of general phenylpropanoid pathway genes, such as *C4H*, *4CL1*, etc. [73]. In Tartary buckwheat, SG4 R2R3-MYB members FtMYB14/15/16 could directly target *FtPAL* gene and inhibit activity of its promoter, thereby negatively regulating rutin biosynthesis [74, 75]. FtMYB11 and FtMYB13 are non-typical R2R3-MYB repressors that do not belong to SG4 R2R3-MYB while their function is similar to that of FtMYB14/15/16 [74, 76]. These SG4 R2R3-MYBs target and negatively regulate the general phenylpropanoid pathway genes.

In addition, AtMYB13 is an SG2 R2R3-MYB and could activate promoters of *AtCHS*, *AtCHI*, and *AtFLS1*, thereby enhancing flavonol accumulation in *Arabidopsis* seedlings [7]*.* In Chinese bayberry, two SG44 R2R3-MYB proteins, MrMYB5 and MrMYB5L, could activate transcription of *MrF3′5′H* and *MrFLS1* by binding to their promoters, based on EMSA and luciferase assays [47]. MrMYB5 or MrMYB5L also interacted with MrbHLH2 to synergistically regulate expression of *MrF3′5′H* and *MrFLS1*, and these MYB-bHLH protein complexes play a crucial role in regulating myricetin biosynthesis [47]. This specific transcriptional mechanism of flavonol biosynthesis might be the reason for the high accumulation of myricetin derivatives in fruit and leaf of Chinese bayberry (Table 1).

### WRKY transcription factors

The WRKY proteins have been shown to be regulators of flavonol biosynthesis, including activators and repressors (Fig. 1b and Table 2). In apple, overexpression of *MdWRKY11* promoted flavonoid accumulation and upregulated expression of *MdF3H*, *MdFLS*, *MdDFR*, *MdANS*, and *MdUFGT* in apple calli [77]. In tobacco, chromatin immunoprecipitation assays and overexpression experiments demonstrated that NtWRKY11b could target and activate promoters of *NtMYB12*, *NtFLS*, *NtGT5*, and *NtUFGT*, thereby inducing flavonol accumulation [78]. Overexpression of *VqWRKY31* from *Vitis quinquangularis* in grape increased accumulation of flavonoids and stilbenes and promoted expression of *VvCHS*, *VvCHI*, *VvDFR*, *VvFLS*, and *VvSTS* (stilbene synthase), which enhanced powdery mildew resistance [79]. Moreover, VvWRKY70 was identified as a transcriptional repressor of flavonol biosynthesis in grape by inhibiting the transcriptional activity of *VvFLS4* and *VvCHS2/3* [80]. Overexpression of *VvWRKY70* caused a reduction of flavonol contents in transgenic grape calli [80]. These WRKY TFs can directly regulate expression of *FLS* genes to participate in regulation of flavonol biosynthesis.

In addition, there were other WRKY activators involved in regulation of flavonol accumulation by activating early genes in flavonoid biosynthesis (Fig. 1b and Table 2). For example, RNAi and overexpression experiments demonstrated that *Arabidopsis* AtWRKY23 functioned as a positive regulator of flavonol biosynthesis by activating *AtF3′H* expression and was also required for proper root growth and development [5]. In cotton (*Gossypium hirsutum*), ChIP–qPCR, yeast two-hybrid, biomolecular fluorescence complementation, and firefly luciferase complementation imaging assays revealed that GhWRKY41could form a homodimer with itself to directly activate GhWRKY41 itself and expression of *GhC4H* and *Gh4CL*, which promoted accumulation of flavonoids and lignin to improve cotton resistance to *Verticillium dahliae* [81]. Overexpression of *VqWRKY56* from *Vitis quinquangularis* increased flavonoid content by directly targeting *VvCHS3* and other flavonoid biosynthetic genes in the transgenic grape leaf, which reduced susceptibility to powdery mildew [82].

### bZIP transcription factors

A well-studied example is *Arabidopsis* ELONGATED HYPOCOTYL 5 (HY5), a bZIP TF that activates expression of *AtCHS*, *AtFLS*, and other genes to regulate flavonoid accumulation during photomorphogenesis in seedlings [83]. In recent years, several newly discovered bZIP activators have also been found to be involved in positive regulation of flavonol biosynthesis (Fig. 1b and Table 2). In *Populus tremula* × *P. alba*, overexpression and suppression experiments indicated that PtabZIP1L could positively regulate flavonoid accumulation by affecting expression of *PtaFLS2/4*, which mediated lateral root development and drought resistance [84]. In grape, CRISPR/Cas9-mediated mutagenesis of *VvbZIP36* promoted anthocyanin accumulation but inhibited flavonol biosynthesis in the leaf, which was associated with upregulation of anthocyanin biosynthetic genes and downregulation of *VvFLS2/4* and two *VvFLR* (flavonol-3-*O*-rhamnosyltransferase) genes, respectively [85]. Overexpression of grape *VvibZIP22* in tobacco promoted accumulation of flavonols and anthocyanins and induced expression of *NtPAL*, *NtCHS*, *NtDFR*, and *NtANS* [86]. In rice, OsbZIP48 was identified as a positive regulator of flavonoid biosynthesis through a metabolite-based genome-wide association study [87]. Yeast one-hybrid and luciferase assays further demonstrated that OsbZIP48 could directly bind to promoters of *Os4CL5* and *OsCHS* and activate their transcription [87]. Interestingly, pear PpbZIP44 could positively regulate expression of *PpF3H* and *PpADT*, which encodes an enzyme, arogenate dehydratase, of primary metabolism as a key determinant of carbon flow into the phenylpropanoid pathway [88]. Therefore, transient overexpression of *PpbZIP44* in pear fruit promoted accumulation of phenylalanine and flavonoids [88].

### AP2/ERF transcription factors

In recent years, several AP2/ERF TFs have been reported to be involved in positive or negative regulation of flavonol biosynthesis (Fig. 1b and Table 2), but it is unknown whether these ERFs are regulated by ethylene signals. In tomato, overexpression of *SlERF.G3-like* activated expression of *SlFLS* and other early genes in the flavonoid biosynthesis pathway, such as *SlCHS1/2*, *SlCHI*, *SlF3H*, and *SlF3′H*, which resulted in induction of flavonol content in fruit [89]. SlERF.G3-like appeared to act independently of the flavonol-specific activator SlMYB12 [89]. Overexpressing *MdAP2-34* in apple callus induced flavonol accumulation by targeting and activating the *MdF3′H* promoter [90]. Overexpression of citrus *CsERF003* in tomato led to accumulation of flavonol glycosides and naringenin chalcone by activating expression of *SlPAL*, *SlC4H*, *Sl4CL*, *SlCHS*, *SlCHI*, *SlF3′H*, and *SlFLS* [91]. In addition, an ERF transcription repressor, FtERF-EAR3, was identified to inhibit *FtF3H* expression and flavonol biosynthesis by binding to the GCC-box in the *FtF3H* promoter in Tartary buckwheat [92].

### Other transcription factors

Members of other TF families have also been reported to be involved in regulation of flavonol biosynthesis and can be divided into two classes (Fig. 1b and Table 2). First, TFs such as MdSCL8, AaYABBY5, and PtHSF5a directly target *FLS* and regulate its expression. In apple, 5-aminolevulinic acid (ALA) inhibited expression of *MdSCL8*, which alleviated its transcriptional repression of *MdFLS1* and promoted flavonol accumulation [93]. In *Artemisia annua*, overexpression of *AaYABBY5* upregulated expression of *AaPAL*, *AaCHS*, *AaCHI*, *AaFLS*, *AaFSII*, *AaLDOX*, and *AaUFGT*, resulting in a significant increase in total flavonoid content [94]. In *Populus tomentosa*, overexpression of *PtHSFA5a* upregulated expression of *PtCHS1*, *PtF3′H2*, and *PtFLS1*/*2*, leading to a significant increase in flavonol content in the transgenic poplar [95]. EMSA, ChIP–qPCR, and luciferase assays demonstrated that PtHSFA5a can directly bind to the promoters of *PtCHS1* and *PtFLS1* to enhance their transcription [95]. Second, TFs act as regulators by targeting early genes in flavonoid biosynthesis. In *Arabidopsis*, REPLUMLESS (RPL) TF was necessary for bacterial resistance and could repress flavonol accumulation by inhibiting expression of the *CHI* gene, which regulated auxin transport to promote plant growth [96]. In tobacco, overexpression and suppression experiments showed that an HD-ZIP IV TF, NtHDG2, could regulate flavonol biosynthesis by targeting and activating promoters of *NtF3′H* and *NtF3GT* [97]. In sweet potato (*Ipomoea batatas*), EMSA and ChIP–qPCR indicated that IbBBX29 could bind to specific T/G-boxes in the promoters of *IbCHS1*, *IbCHI1*, and *IbF3′H* to activate their expression [98]. Overexpression of *IbBBX29* increased contents of flavonols and other flavonoids by upregulating expression of flavonoid biosynthetic genes in storage roots of sweet potato [98].

**Figure 2:**
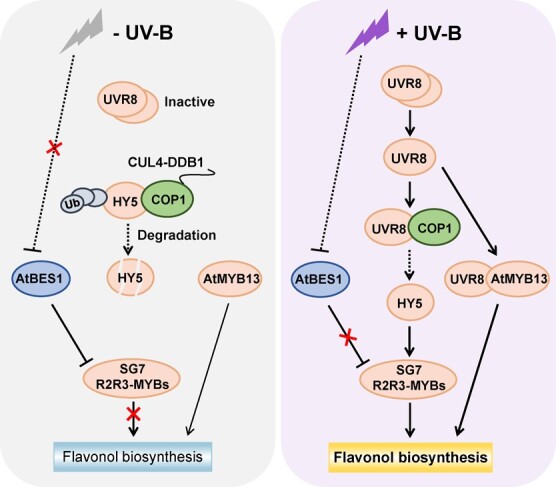
Cascade transcriptional regulation of flavonol biosynthesis in plants under UV-B radiation. The yellow box with bold letters means promotion of flavonol biosynthesis, whereas the blue box with non-bold letters represents repression of flavonol biosynthesis. Line thickness indicates the activity of transcriptional activation or repression. Light orange and blue-gray ovals represent activators and repressors, respectively.

## Cascade transcriptional regulation of flavonol biosynthesis

### Post-transcriptional regulation

miRNAs are a class of non-coding RNAs with lengths 20–24 nt and regulate post-transcriptional processes by recognizing target genes through base complementarity, leading to mRNA cleavage or translational inhibition [99]. With the application and deep exploration of genomics, several miRNAs involved in flavonol biosynthesis have been identified in plants (Fig. 1b and Table 2). The miR858-MYB regulatory modules have been reported to regulate flavonol biosynthesis in plants. In *Arabidopsis*, transgenic experiments indicated that AtmiR858a directly targeted and cleaved SG7 *R2R3-MYB* genes, including *AtMYB11/12/111*, resulting in the negative regulation of flavonol biosynthesis [100]. Subsequent research revealed that primary AtMIR858a encoded a small peptide, miPEP858a, which was involved in transcriptional regulation of AtmiR858a [101]. This peptide could negatively regulate flavonol biosynthesis by inhibiting expression of *AtMYB12* through AtmiR858a. In potato, overexpression of StmiR858 inhibited expression of *StMYB12A/C* genes, leading to a reduction in flavonol accumulation [15]. As research goes on, members of other miRNA families and their different target genes are continually discovered. In cotton, overexpression of *GhSPL10*, a target of GhmiR157a, resulted in a significant increase in flavonol accumulation, which promoted initial cellular dedifferentiation and callus proliferation [102]. In *Arabidopsis*, overexpression of apple MdmiR172 targeting *MdAP2_1a* reduced levels of anthocyanins and flavonols as well as expression of *AtFLS1* and other flavonoid biosynthetic genes in plantlets, which may be caused by regulation of the MdAP2_1a-MdMYB10 module [103]. Current research on miRNAs regulating flavonol biosynthesis is quite limited, warranting further investigation.

### UV-B regulation

Solar ultraviolet (UV) light consists of UV-A (320–400 nm) and a portion of UV-B (280–320 nm). Elevated UV-B radiation leads to the generation of numerous free radicals within plants, which induces damage to DNA, RNA, and proteins. Flavonols serve as efficient scavengers of free radicals and UV-B absorbers [4]. Preharvest UV-B radiation was found to increase flavonol accumulation in apple and grape fruits [104, 105]. In many studies UV-B radiation as a method of postharvest treatment was applied to improve flavonol content in vegetables and fruits, such as apple [106], asparagus [107], broccoli [108], Chinese bayberry [47], cucumber [109], kale (*Brassica oleracea* var. *sabellica*) [110], mango [111], onion [112], peach [35], and tomato [113]. Recently, significant progress has been made in the regulatory mechanisms of plant flavonol biosynthesis in response to UV-B signal in UVR8-dependent and UVR8-independent ways, including R2R3-MYB and other non-MYB TFs (Fig. 2 and Table 2).

*UVR8-dependent UV-B signaling pathway*. UV-B signal is perceived by the photoreceptor UV RESISTANCE LOCUS (UVR8), which is a plant-specific and highly conserved protein [114, 115]. UVR8 was inactive in its dimeric form in the absence of UV-B, and CONSTITUTIVELY PHOTOMORPHOGENIC 1 (COP1) induced ubiquitination and degradation of ELONGATED HYPOCOTYL 5 (HY5) by the 26S proteasome, thus repressing expression of downstream target genes [114, 116] (Fig. 2). After UV-B perception, dimeric UVR8 underwent monomerization to form monomeric UVR8, which interacted with COP1 to form a complex that repressed COP1 activity [114, 116]. The central TF HY5 was ultimately stabilized and activated transcription of UV-B-responsive genes [116, 117].

**Figure 3:**
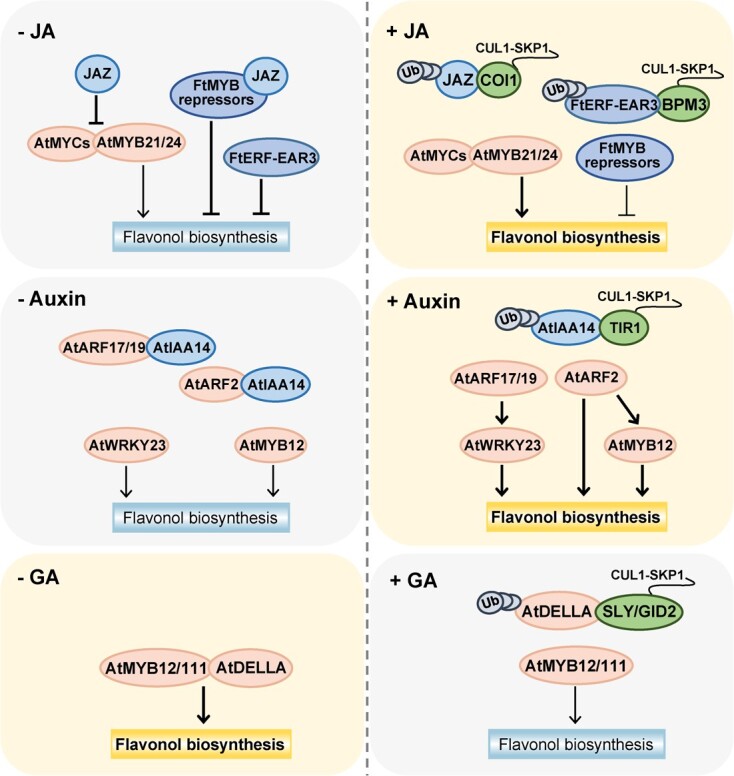
Cascade transcriptional regulation of flavonol biosynthesis in plants in response to different hormonal signals. Yellow boxes with bold letters mean promotion of flavonol biosynthesis, whereas blue boxes with non-bold letters represent repression of flavonol biosynthesis. Line thickness indicates the activity of transcriptional activation or repression. Light yellow and light gray backgrounds indicate activation and repression of flavonol biosynthesis, respectively. Light orange and blue-gray ovals represent activators and repressors, respectively. JA, jasmonates; GA, gibberellic acid; Ub, ubiquitin.

**Table 3:** Different regulation modules in cascade transcriptional regulation of flavonol biosynthesis in plants.

**Regulation module**	**Species**	**Representative genes and miRNAs**	**Function**	**References**
miR858-SG7 R2R3-MYB	*Arabidopsis thaliana*, *Solanum tuberosum*	AtmiR858, StmiR858	Repressor	15, 100, 101
miRNA157-SPL10	*Gossypium hirsutum*	GhmiR157a	Repressor	102
miRNA172-AP2	*Malus domestica*	MdmiR172	Repressor	103
UVR8-COP1-HY5	*Arabidopsis thaliana*, *Malus domestica*	*AtUVR8*, *AtHY5*, *MdHY5*	Activator	4, 7, 104
UVR8-MYB13	*Arabidopsis thaliana*	*AtUVR8*	Activator	7
BES1-SG7 R2R3-MYB	*Arabidopsis thaliana*	*AtBES1*	Repressor	120
SCF^COI1^-JAZ	*Fagopyrum tataricum*	*FtJAZ1*	Repressor	74, 76
SCF^BPM3^-ERF	*Fagopyrum tataricum*	*FtERF-EAR3*	Repressor	92
SCF^TIR1^-Aux/IAA-ARF	*Arabidopsis thaliana*	*AtARF2/17/19*	Activator	5, 131, 132
SCF^GID2^-DELLA- SG7 R2R3-MYB	*Arabidopsis thaliana*	*AtDELLA*	Activator	135
PP2A-SnRK2.6	*Malus domestica*	*MdPP2AC*	Protein dephosphorylation	140
MYB4-SG7 R2R3-MYB	*Arabidopsis thaliana*	*AtMYB4*	Repressor	141
IAA-HSF	*Populus tomentosa*	*PtIAA17.1*	Repressor	95
SBP-MYB12	*Solanum lycopersicum*	*SlSPL-CNR*	Repressor	142
RLCK-bZIP	*Oryza sativa*	*OsRLCK160*	Protein phosphorylation	87

In *Arabidopsis*, HY5 could directly target and activate expression of *AtMYB12* and *AtMYB111* under UV-B radiation, leading to flavonol accumulation in the seedlings [4, 118] (Fig. 2). This HY5-SG7 R2R3-MYB regulatory module has also been identified in horticultural crops such as grape [119], apple [104], and tea [45]. MdHY5 and MdMYB22 (a homolog of AtMYB12) from apple could also synergistically regulate transcription of *MdCHS* and *MdFLS*, leading to the induction of flavonol accumulation under UV-B radiation [104]. These results indicated that HY5 regulates flavonol biosynthesis in two steps. First, it directly binds to promoters of SG7 *R2R3-MYB*s and induces their expression. Secondly, it can interact with SG7 R2R3-MYBs to synergistically regulate expression of *FLS* and other early genes in flavonoid biosynthesis. In addition to the HY5-SG7 R2R3-MYB module, monomeric AtUVR8 in *Arabidopsis* could also directly interact with AtMYB13 to form a complex that enhanced the affinity of AtMYB13 for promoters of *AtCHS*, *AtCHI*, and *AtFLS1* [7] (Fig. 2). This UVR8-MYB13 module further promoted flavonol accumulation and plant resistance to UV-B stress [7].

*UVR8-independent UV-B signaling pathway*. Apart from the UVR8-dependent UV-B signaling pathway, the UVR8-independent stress response has been recently identified in plants (Fig. 2 and Table 2). Under white light conditions without UV-B radiation, the brassinosteroid signal led to activation of *BRI1-EMS-SUPPRESSOR 1* (*BES1*) (a master TF in brassinosteroid signal transduction) in *Arabidopsis* [120]. This activation of *AtBES1* resulted in downregulation of *AtMYB11*, *AtMYB12*, and *AtMYB111* expression, consequently leading to a decrease in flavonol accumulation [120]. However, when *Arabidopsis* plants were exposed to UV-B radiation, the UVR8-COP1-HY5 module was activated to initiate UV-B photomorphogenesis, including activation of SG7 *R2R3-MYB* expression [120]. In addition, UV-B stress could also inhibit expression of *AtBES1* in a UVR8-independent manner, which removed inhibition of SG7 *R2R3-MYB* expression and promoted flavonol accumulation [120]. The UV-B stress-induced inhibition of *AtBES1* expression reallocated more energy towards flavonol biosynthesis, which promptly shifted plants from brassinosteroid-promoted growth to UV-B stress response and ensured normal plant growth under adverse conditions.

### Phytohormonal regulation

*Jasmonates*. Jasmonates (JAs) are vital plant hormones and trigger a cascade of stress-related gene expressions in response to biotic and abiotic stress through the signaling module of the SCF^COI1^-jasmonate-ZIM domain (JAZ). Induction of flavonol accumulation by JAs has been observed in plants such as blackberry (*Rubus* sp.) [121], *G. biloba* [122], and Tartary buckwheat [76]. In *Arabidopsis*, JAZ proteins interfered with MYB-bHLH complexes, which were composed of IIIe-bHLH TFs (MYC2, MYC3, MYC4, and MYC5) and SG19 R2R3-MYB TFs (MYB21 and MYB24) [123]. JA signals were perceived by CORONATINE-INSENSITIVE PROTEIN 1 (COI1), which recruited proteins to the Skp1/Cullin/F-box (SCF^COI1^) complex for ubiquitination and subsequent degradation by the 26S proteasome pathway [124, 125]. Thus, the degradation of JAZ proteins led to release of the MYB-bHLH complexes, which regulated expression of downstream genes involved in stamen development [123, 126] (Fig. 3). A further study demonstrated that AtMYB21 and AtMYB24 activated transcription of *AtFLS1* to induce accumulation of pollen-specific flavonols, which enhanced reactive oxygen species (ROS) scavenging capacity and contributed to male fertility [71].

Transcriptional repressors including R2R3-MYBs and AP2/ERFs also respond to JA signaling and participate in regulation of flavonol biosynthesis (Fig. 3 and Table 2). On the one hand, JA-responsive FtMYB11/13/14/15 repressors directly inhibited expression of the *FtPAL* gene, and FtJAZ protein interacting with these R2R3-MYB repressors enhanced their activity of transcriptional repression [74, 76]. On the other hand, JAs activated and stabilized the CUL3 BTB/POZMATH (BPM) E3 ligase FtBPM3, which mediated the 26S proteasomal pathway to induce degradation of these R2R3-MYB repressors and FtJAZ, thereby relieving their inhibition of the *FtPAL* gene [74, 127]. Furthermore, the JA-stabilized FtBPM3 also interacted with FtERF-EAR3 and mediated degradation of FtERF-EAR3, which alleviated transcriptional repression of FtERF-EAR3 on the *FtF3H* gene and induced flavonol accumulation in Tartary buckwheat [92].

*Auxin*. Auxin is an important plant hormone for plant growth and development through the SCF^TIR1^-IAA-ARF module [128]. Auxin has also been reported to positively regulate flavonol biosynthesis [129, 130]. In *Arabidopsis*, Lewis *et al*. [130] found that auxin could upregulate expression of *AtMYB12*, *AtCHS*, *AtCHI*, *AtF3′H*, and *AtFLS* through the Transport Inhibitor Response1 (TIR1) signaling pathway [130]. They speculated that auxin response factors (ARFs) play a crucial role in this process [130] (Fig. 3). Recently, AtARF2 was identified as a positive regulator of flavonol biosynthesis through directly activating transcription of the *AtMYB12* and *AtFLS* genes [131]. Another study indicated that auxin-induced flavonol accumulation also depended on the ARF pathway [5] (Fig. 3 and Table 2). In *Arabidopsis*, auxin may mediate degradation of SOLITARY ROOT/INDOLE-3-ACETIC ACID14 (SLR/IAA14) by the SCF^TIR1^ complex, which led to release of AtARF7/19 and subsequent induction of *AtWRYY23* expression, thereby activating *AtF3′H* expression to induce flavonol biosynthesis in the roots [5, 132].

*Gibberellic acid*. Gibberellic acid (GA) is an important plant hormone for plant growth and participates in negative regulation of flavonol biosynthesis through the GID1-SCF^SLY1/GID2^-DELLA signaling module [133–135]. In plants, GA signals promoted the interaction between GID1 (GA-INSENSITIVE DWARF1) and DELLA proteins, enhancing the binding affinity of the GID1-DELLA complex with the SCF^SLY1/GID2^ complex. This interaction led to the degradation of DELLA proteins by the 26S proteasome pathway [133, 134] (Fig. 3). Recently, a study in *Arabidopsis* revealed that GA negatively regulated flavonol biosynthesis through the DELLA-SG7 R2R3-MYB module [135] (Fig. 3 and Table 2). In the absence of GA, DELLA protein accumulated and physically interacted with SG7 R2R3-MYBs, which enhanced the transcriptional activation activity of AtMYB12 and AtMYB111 on promoters of *AtFLS1* and *AtF3H* [135]. This promoted flavonol biosynthesis in *Arabidopsis* roots and inhibited auxin transport and root growth. Conversely, GA signaling promoted degradation of the DELLA protein by the 26S proteasome pathway. Subsequently, this reduced the transcriptional activation activity of SG7 R2R3-MYB proteins and flavonol content in *Arabidopsis* roots, which led to an increase of auxin accumulation in root tip cells and promotion of root growth [135].

*Abscisic acid*. Abscisic acid (ABA) as a plant hormone plays an important role in plant growth and fruit ripening [136]. ABA can also modulate the stomatal aperture by directly promoting production of ROS in plant guard cells [137]. Flavonols, as important ROS scavengers, are involved in the regulation of ABA-induced stomatal closure in plants [138, 139]. In tobacco, ABA treatment could inhibit expression of *NtMYB184* (a flavonol-specific activator), which reduced production of flavonols and thus increased ROS levels to regulate stomatal closure [65]. ALA, known as a new natural plant growth regulator, can reverse ABA-induced stomatal closure [140]. In apple, ALA treatment enhanced protein abundance and phosphorylation of protein phosphatase 2AC (MdPP2AC), which promoted the interactions of different PP2A subunits and increased holoenzyme activity [140]. Phosphorylated PP2A interacted with and dephosphorylated MdSnRK2.6 (sucrose non-fermenting 1-related protein kinase 2.6), which induced flavonol accumulation and thus reduced ROS levels in the guard cells to open stomata [140]. In addition, ALA treatment could inhibit expression of *MdSCL8* (a flavonol repressor), which may promote expression of *MdFLS1* and flavonol accumulation to participate in regulation of stomata opening [93].

*Other regulation modules*. Several members of other TF families were identified as repressors by negatively regulating transcriptional activity of flavonol-related activators (Table 3). In *Arabidopsis*, AtMYB4 could repress activation of the *AtCHS *and *AtFLS *promoters by AtMYB12 and AtMYB111 [141]. In tomato, loss-of-function and luciferase assays indicated that SlSPL-CNR functioned as a negative regulator of flavonol biosynthesis by repressing SlMYB12 transcription activity [142]. In *P. tomentosa*, yeast two-hybrid, pull-down, co-immunoprecipitation, and luciferase assays demonstrated that PtIAA17.1 could interact with PtHSFA5a to suppress PtHSFA5a-mediated activation of *PtCHS1* and *PtFLS1* [75]. Salt stress enhanced the stability of PtIAA17.1, resulting in the promotion of its interaction with PtHSFA5a and the repression of flavonol biosynthesis [95]. In addition, a receptor-like kinase (OsRLCK160) could regulate flavonoid accumulation in rice by interacting with and phosphorylating OsbZIP48 [87].

## Conclusions and perspective

Flavonols are an important branch of the flavonoids with excellent bioactive activities, and they are abundant in horticultural crops. Due to the broad function of flavonols in plants, sophisticated regulation networks involving different types of TFs (activators and repressors) and miRNAs have evolved, illustrating fine-tuned flavonol homeostasis under specific environmental conditions or hormonal signals. Significant progress has been achieved in unraveling the transcriptional regulation of flavonol biosynthesis in *Arabidopsis* and some horticultural crops, such as tea, apple, and Chinese bayberry. However, more flavonol-rich horticultural crops deserve in-depth investigations to discover novel TFs and elucidate the specific regulatory mechanisms of flavonol biosynthesis. Besides transcriptional regulation, post-transcriptional regulation, post-translational modifications, and epigenetic regulation of flavonol biosynthesis in plants are also worthy of further exploration.

In addition, flavonol derivatives are associated with the astringency of horticultural crops. Increasing the flavonol content of fruits and vegetables by genetic selection, genetic engineering, or physical treatment may be of interest for human health, but it might negatively affect the taste quality. Structural modification of secondary metabolites caused by decorations such as hydroxylation and glycosylation can alter the taste of the compounds. So far, there is limited research reporting the improvement of the flavor quality of flavonols by structural modification, which deserves future study. This will provide valuable insights for utilizing molecular design breeding and synthetic biology to enhance flavonol accumulation in horticultural crops with significant bioactivities and unaffected flavor quality, thereby facilitating the development of the horticultural industry.

## Acknowledgements

This work was supported by the National Natural Science Foundation of China (32372667), the Natural Science Foundation of Shandong Province (ZR2023QC228), the Key Research and Development Program of Zhejiang Province (2021C02001), and the Key Project for New Variety Breeding in Agriculture of Zhejiang Province (2021C02066-3).

## Author contributions

Y.C. and X.L. designed this review. Y.C., Y.M., R.Z., and Z.Z. conducted the literature review and wrote the manuscript. K.C., C.X., and X.L. carefully compiled and revised the paper. X.Y. provided discussion and comments on the paper. All authors approved the final submission.

## Data availability

The authors confirm that all data in this study are available and can be found in this article.

## Conflict of interest

The authors declare that they have no conflict of interest.
